# Plasma interleukin-8 as a predictive biomarker for tyrosine kinase inhibitor response in advanced hepatocellular carcinoma

**DOI:** 10.1007/s12672-026-05055-4

**Published:** 2026-05-11

**Authors:** Sujin Kim, Hye Ri Ahn, Hui Gyeong Kim, Dooyeon Kim, Ji Eun Han, Soon Sun Kim, Jae Youn Cheong, Jung Woo Eun, Hyo Jung Cho

**Affiliations:** 1 Department of Gastroenterology, Ajou University School of Medicine, Suwon, South Korea; 2 Department of Biomedical Sciences, Ajou University Graduate School of Medicine, Suwon, South Korea; 3 BK21 R&E initiative for Advanced Precision Medicine, Ajou University School of Medicine, Suwon, South Korea

**Keywords:** Hepatocellular carcinoma, Tyrosine kinase inhibitors, Olink proteomics, Plasma, Biomarkers, Interleukin-8

## Abstract

**Background and aim:**

Hepatocellular carcinoma (HCC) is the predominant primary liver cancer and third leading cause of cancer-related death. Despite improvements in overall survival (OS), those in HCC-specific survival rates remain modest. Tyrosine kinase inhibitors (TKIs) are crucial first-line HCC treatments when immune checkpoint inhibitors are unsuitable. To identify plasma biomarkers predicting the therapeutic response to TKIs in patients with HCC.

**Methods:**

Pre-treatment plasma samples from 60 patients with advanced HCC treated with sorafenib or lenvatinib were analyzed using targeted proteomics. Differentially expressed proteins were identified based on the treatment response (*P* < 0.05).

**Results:**

Plasma levels of metalloproteinase 12 and vascular endothelial growth factor A were elevated in patients with progressive disease compared with those with partial response or stable disease, correlating with shorter progression-free survival (PFS) and OS. Among patients with PFS ≥ 12 months, C-C motif chemokine ligand 20, C-X-C motif chemokine ligand 1, C-X-C motif chemokine ligand 5, fibroblast growth factor 2, interleukin (IL)-7, IL-8, IL-18, latency-associated peptide transforming growth factor beta 1, and mucin 16 expressions were significantly upregulated, with IL-8 (CXCL8) levels demonstrating the highest predictive accuracy (area under the receiver operating characteristic = 0.91) and prognostic power for PFS (hazard ratio; HR = 2.97, *P* = 0.0015) and OS (HR = 3.64, *P* = 0.001). *CXCL8* expression was predominantly localized in tumor-associated myeloid cells and enriched in epithelial–mesenchymal transition- and immune modulation-related pathways, highlighting its importance in the tumor microenvironment.

**Conclusions:**

Elevated plasma IL-8 levels are strongly associated with poor outcomes in patients with advanced HCC undergoing TKI treatment, suggesting a potential role for IL-8 in guiding TKI therapeutic decisions and identifying high-risk patients.

**Supplementary Information:**

The online version contains supplementary material available at 10.1007/s12672-026-05055-4.

## Introduction

Primary liver cancer is the third leading cause of cancer-related death worldwide, and hepatocellular carcinoma (HCC) accounts for approximately 80% of all cases [1, 2]. Despite advancements in overall survival (OS), improvements in HCC-specific survival rates remain modest. The five-year survival rate for advanced HCC is only 2–11%, primarily due to its frequent diagnosis at a stage where curative treatments are no longer effective [3]. In recent years, immune checkpoint inhibitor (ICI)-based therapies, such as the combination of atezolizumab and bevacizumab, have been established as first-line treatments. Tyrosine kinase inhibitors (TKIs), including sorafenib and lenvatinib, remain crucial first-line options when ICI-based regimens are unsuitable, and are used as second-line treatments for patients who fail to respond to ICI-based therapies [4–6].

Protein tyrosine kinases catalyze the transfer of the γ-phosphate of ATP to the tyrosine residues of protein substrates [7]. Activated tyrosine kinases drive tumor cell proliferation and growth, inhibit apoptosis, and promote angiogenesis and metastasis [8, 9]. As these effects are initiated by receptor tyrosine kinase activation, treatment with TKIs is the primary targeted therapy. TKIs are widely used to treat non-small-cell lung cancer [10], renal cell carcinoma [11], gastrointestinal stromal tumors [12], and breast cancer [13]. Sorafenib and lenvatinib are key TKIs in the treatment of advanced-stage HCC [14, 15]. However, their low response rates remain a significant challenge, with approximately 10–20% of patients achieving an objective response [16, 17]. Consequently, a substantial proportion of patients treated with tyrosine kinase inhibitors experience treatment-related toxicities without meaningful clinical benefit, underscoring the critical need for predictive biomarkers to guide treatment decisions and optimize patient selection in HCC [18–20] Reliable biomarkers may enable clinicians to tailor therapy more effectively and identify the patients most likely to benefit from TKI treatment [21, 22]. Several studies have suggested that the immunosuppressive tumor microenvironment (TME) plays a crucial role in influencing the TKI treatment response [23–25]. The immunosuppressive nature of the TME is closely associated with the expression of circulating plasma proteins, which may serve as valuable biomarkers for predicting therapeutic outcomes.

To identify plasma biomarkers associated with TKI efficacy in patients with advanced HCC, we conducted a targeted proteomic analysis of pre-treatment blood samples, focusing on 96 key immuno-oncology proteins involved in immune regulation and TME interactions. By analyzing these immune-related proteins, we aimed to improve patient stratification and guide treatment planning, ultimately enhancing clinical outcomes in patients with advanced HCC.

## Methods

### Study design and patient enrollment

We measured the expression of 96 immuno-oncology panel proteins in pre-treatment plasma samples from patients with advanced HCC treated with TKIs. The predictive power of the identified DEPs was assessed using receiver operating characteristic (ROC) curve analysis, and survival analysis was performed based on their expression.

Blood samples and clinical data from patients with advanced HCC treated with TKIs were obtained from the Biobank of Ajou University Hospital (Suwon, South Korea), a single tertiary referral center in an HBV-endemic region and a member of the Korea Biobank Network. The inclusion criteria were as follows: (1) age between 18 and 80 years; (2) modified Union for International Cancer Control (mUICC) stage IVa or IVb [26]; (3) mUICC stage II or III with extensive disease or main vascular invasion precluding curative local treatment; (4) treatment with sorafenib or lenvatinib for more than 4 weeks; and (5) availability of plasma samples collected within one month before treatment initiation. Patients were excluded if they had a history of malignancy other than HCC or if data for evaluating the treatment response after TKI administration were insufficient. All patients had underlying cirrhosis, which was defined based on established clinical criteria, including clinical and laboratory features such as thrombocytopenia as a surrogate marker of portal hypertension, in accordance with international guidelines (Supplementary Table S1).

The clinical data included patient demographics (age and sex), administration of TKIs (sorafenib or lenvatinib), laboratory parameters (platelet count, albumin, bilirubin, creatinine, alpha-fetoprotein, alanine aminotransferase, and aspartate aminotransferase levels; and international normalized ratio), etiologies of underlying liver disease (chronic hepatitis B, chronic hepatitis C, alcoholic liver disease, and unknown causes, with chronic hepatitis B being the predominant etiology in this cohort; Supplementary Table S1), tumor stage (mUICC stage), treatment response to TKIs, and survival outcomes, including progression-free survival (PFS) and OS.

The study protocol was reviewed and approved by the Institutional Review Board of Ajou University Hospital, Suwon, South Korea (AJIRB-BMR-SMP-17-188), which also waived the requirement for informed consent due to the retrospective design of the study and the use of anonymized biobank samples.

### Definition of terms

HCC was diagnosed in accordance with the guidelines of the American Association for the Study of Liver Diseases [27]. Tumor stage was defined according to the mUICC staging system [26]. Tumor treatment response was evaluated according to the modified Response Evaluation Criteria in Solid Tumors (mRECIST). The criteria were as follows: complete response (CR), characterized by the disappearance of all intratumoral arterial enhancements in the target lesions. Partial response (PR) was defined as a reduction of at least 30% in the sum of the diameters of the viable (arterially enhancing) target lesions, using the baseline sum of diameters as the reference. Stable disease (SD) referred to cases that did not meet the criteria for either PR or progressive disease (PD). PD was defined as an increase of at least 20% in the sum of the diameters of the viable target lesions compared to the smallest recorded sum after treatment [28]. PFS was defined as the time from the initiation of TKI treatment to the date of disease progression or death from any cause, whichever occurred first. OS was defined as the time from the initiation of TKI treatment to the date of death from any cause. Patients without disease progression or death at the time of analysis were censored at the date of their last follow-up.

### Plasma sample processing and proteomic analysis using the Olink^®^ target 96 immuno-oncology panel

Pre-treatment plasma samples from patients with advanced HCC treated with TKIs (*n* = 60) were analyzed using the Olink^®^ Target 96 Immuno-Oncology panel, which is based on a high-sensitivity and specific proximity extension assay technology (Olink Proteomics, Uppsala, Sweden) that can simultaneously analyze 96 immuno-oncology-related protein biomarkers. The Olink^®^ Target 96 panel process was conducted following the manufacturer’s protocol. Plasma samples (1 µL) were incubated overnight at 4 °C with 92 antibody pairs, each conjugated to DNA tags, to allow binding to the target proteins. The next day, extension and amplification steps were performed, where oligonucleotides brought into proximity were hybridized and extended using DNA polymerase to create DNA barcodes. These barcodes were amplified using PCR with the following thermocycling conditions: 50 °C for 20 min, 95 °C for 5 min, followed by 17 cycles of 95 °C for 30 s, 54 °C for 1 min, and 60 °C for 1 min, with a final step at 10 °C. The DNA reporters for each biomarker were then quantified using high-throughput real-time qPCR on the Olink^®^ Signature Q100 system. Raw data from the Olink^®^ Signature Q100 were imported, quality-checked, and normalized using inter-plate control to obtain normalized protein eXpression (NPX) values via Olink NPX Signature software (1.11.0). Quality control-flagged samples with deviations from the median NPX values of the internal controls and assays were filtered based on the limit of detection. DEPs were identified using a paired *t-*test with a *P*-value < 0.05, and false discovery rate control was performed using the Benjamini–Hochberg method. Data analysis and visualization were conducted using R version 4.2.2, with the OlinkAnalyze R package for the statistical analysis and visualization of DEPs.

### DEP analysis

DEP analysis was performed to identify differentially expressed plasma proteins among three comparison groups: PR versus SD/PD, PR/SD versus PD, and disease control (DC, PFS ≥ 12 months) vversus disease progression (DP). The predictive value of significant proteins was assessed using ROC curve analysis, and Kaplan–Meier survival analysis was conducted to evaluate their association with clinical outcomes, including PFS and OS.

### Public data acquisition

Publicly available omics datasets were obtained from Gene Expression Omnibus (GSE94550, GSE248764, GSE273819, GSE140202, GSE109211, and GSE149614), GepLiver [29], and Mendeley (skrx2fz79n) Data. These datasets include transcriptomic data from TKI-treated HCC cell lines, HCC patient samples, and spatial transcriptomic data from HCC tissue sections. The analysis focused on evaluating C-X-C motif chemokine ligand 8 (CXCL8, also known as interleukin (IL)-8) expression patterns in relation to TKI resistance, liver disease progression, and the spatial localization of malignant versus non-malignant hepatocytes.

### Comprehensive liver dataset analysis

Transcriptomic data from TKI-treated cell lines, including Huh-7, HepG2, and PLC/PRF/5, were analyzed to compare *CXCL8* expression between the TKI-sensitive (Sorafenib-sensitive, Sor-Sen) and TKI-resistant (Sorafenib-resistant, Sor-Res; Lenvatinib-resistant, Len-Res) groups. Similarly, transcriptomic data from patients with HCC were used to compare *CXCL8* expression between sorafenib non-responder (Sor-NR) and responder (Sor-R) groups. Additionally, transcriptomic data from the GepLiver database were used to analyze *CXCL8* expression across various liver conditions, including normal liver, non-alcoholic fatty liver disease, cirrhosis, adjacent HCC (ADJ_HCC), and HCC tissues. Data were normalized to log_2_-transformed expression values and Z-score normalization was applied for consistent visualization. Statistical tests were performed to evaluate the differences in *CXCL8* expression under different conditions.

### Spatial transcriptomics analysis

Spatial transcriptomic analysis was performed using Mendeley Data, which included annotated tissue sections (P7T–P11T) from patients with HCC. Tissue sections were classified into malignant and non-malignant hepatocytes, and CXCL8 expression was visualized using a color gradient representing minimal (blue) to maximal (red) expression. The proportion of *CXCL8* + cells in malignant versus non-malignant hepatocytes was quantified for each tissue section to determine spatial distribution patterns. Differences in *CXCL8* + cells between malignant and non-malignant regions were statistically evaluated, revealing the enrichment of *CXCL8* + cells in malignant hepatocytes.

### Analysis of *CXCL8* expression in single-cell RNA-Seq data from HCC patient samples

Single-cell RNA sequencing (scRNA-seq) data from the GSE149614 dataset were analyzed, focusing on HCC07 and HCC08 patient samples, including normal tissue (NT), primary tumor (PT), and portal vein tumor thrombus (PVTT). Raw data were processed using the Seurat package (v4.0.6) in R. Uniform manifold approximation and projection (UMAP) was used to visualize the distribution of cells, with cell clusters identified through a shared nearest-neighbor graph-based clustering approach and resolution parameters optimized to capture distinct cell populations. Cell-type annotations were assigned using canonical markers for B cells, endothelial cells, fibroblasts, hepatocytes, myeloid cells, and T/NK cells. Differential gene expression analysis was conducted to compare cell clusters in NT, PT, and PVTT groups, focusing on *CXCL8* expression.

Further analysis was performed on myeloid cells in the PT and PVTT. Sub-clustering of myeloid cells was conducted, followed by the identification of the C1, C2, and C3 clusters. Gene set enrichment analysis (GSEA) was performed using the ssGSEA algorithm to assess pathway enrichment in *CXCL8*-positive cells within C1 and C2 clusters. Pathway databases included hallmark gene sets, focusing on epithelial–mesenchymal transition (EMT), inflammatory response, and tumor necrosis factor (TNF)-α signaling via nuclear factor (NF)-κB.

### Correlation of *CXCL8* with EMT-associated genes in TCGA-LIHC cohort

Correlation analysis was conducted to assess the relationship between *CXCL8* expression and EMT-associated genes within *CXCL8* + cells in C1 and C2 clusters, where ssGSEA analysis showed the highest enrichment for EMT pathways. EMT-related genes, including *MMP2*, *MMP9*, *SNAI1*, and *VIM*, were selected for comparison. Bulk RNA-seq data from the The Cancer Genome Atlas (TCGA)-LIHC cohort (*n* = 371) were used for this analysis. Gene expression values were log_2_-transformed, and Pearson correlation coefficients (r) were calculated between *CXCL8* and the selected EMT markers. The significance of correlations was determined using a two-tailed test, with values of *P* < 0.05 considered statistically significant. Scatter plots were generated to visualize the correlations, with regression lines fitted to depict trends in the data.

### Statistical analysis

The statistical significance of the differences between the two groups was assessed using either the paired Student’s *t*-test or the unpaired Welch’s *t*-test using GraphPad Prism (version 9.0; GraphPad Software, San Diego, CA, USA). ROC analysis was conducted using the IBM SPSS software (IBM SPSS Statistics for Windows, version 22.0, released 2013, IBM, Armonk, NY, USA). ROC curves were analyzed to evaluate the diagnostic accuracy of candidate biomarkers. These curves were analyzed for sensitivity and specificity of the candidate biomarkers at various threshold values, and the area under the ROC curve (AUROC) was evaluated with 95% confidence intervals (CIs). Statistical significance was set at *P* < 0.05. Kaplan–Meier survival curves are statistical methods used to estimate survival function, and prognostic changes based on the expression of candidate markers were analyzed. The significance of *P*-value differences between the survival curves was evaluated using the log-rank test, with a *P*-value < 0.05 considered statistically significant.

## Results

### Baseline characteristics of the included patients

**Fig. 1:**
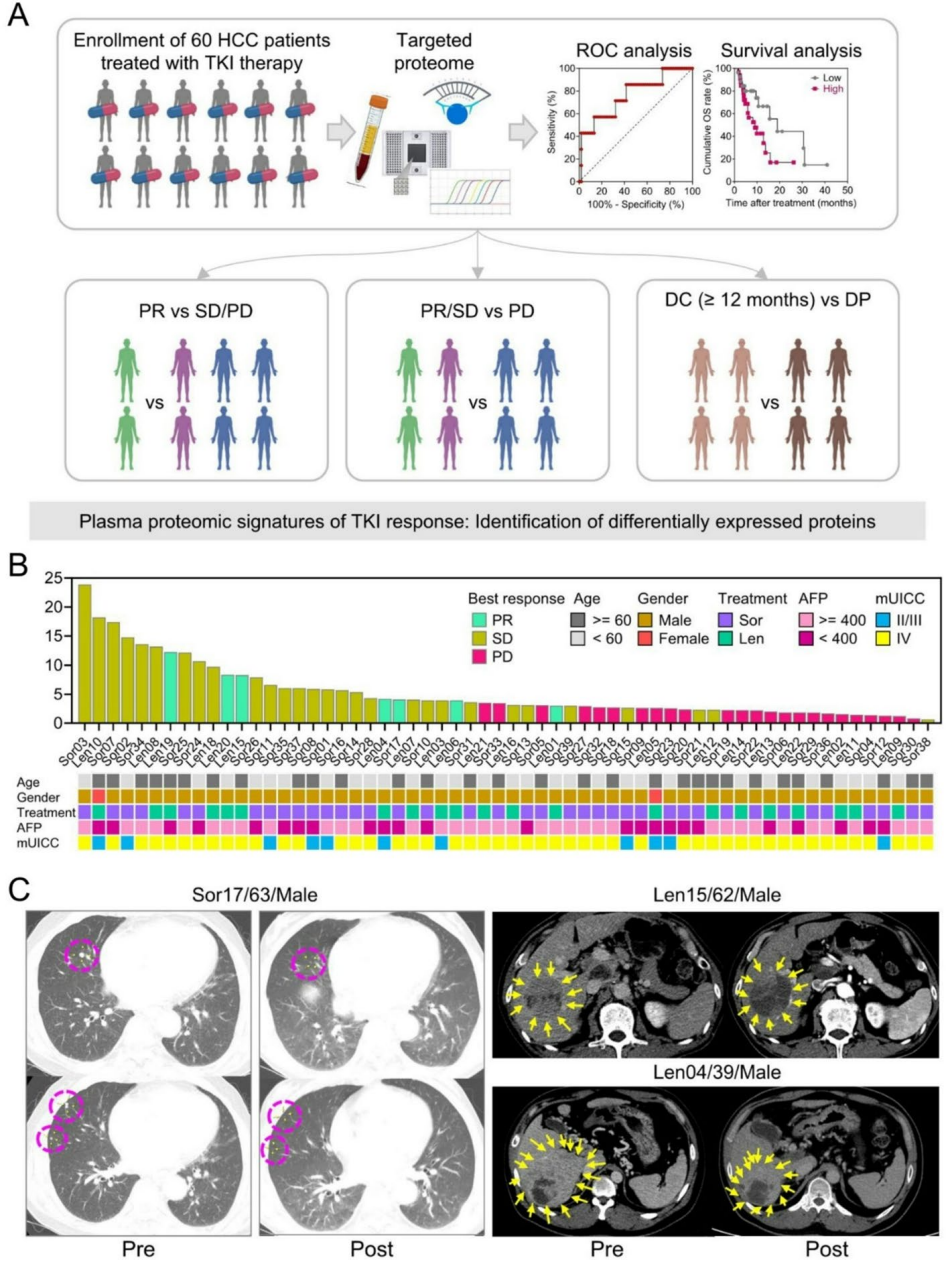
Analysis of molecular profiles and tumor response to tyrosine kinase inhibitor (TKI) therapy in patients with hepatocellular carcinoma (HCC). **a** Flowchart of patient enrollment and study design. **b** Waterfall plot illustrating tumor size reduction in individual patients following TKI therapy. Each bar represents a patient, and the colors correspond to different molecular and clinical features. Heatmap of genomic and clinical features across the patient cohort. The rows indicate individual features, whereas columns represent patients, grouped by treatment response. **c** Representative Computer tomography (CT) images of tumor lesions in the lung (left) and liver (right) before and after TKI therapy. Circles (purple) and arrows (yellow) highlight the tumor boundaries, demonstrating the reduction in tumor size. DC; disease control; DP; disease progression

In a cohort of 60 patients, 39 received sorafenib and 21 received lenvatinib (Fig. 1). Each bar represents a patient, and the color of the bar indicates the degree of drug response. None of the patients demonstrated CR to TKI therapy. However, seven patients had a PR to treatment, 29 had SD, and 24 had PD. The distribution of patients aged older than 60 years and younger than 60 years was equal, with each group consisting of 30 individuals; the proportion of male patients was higher than that of female patients. Forty-nine patients (18.3%) had mUICC stage IV disease (Fig. 1a). Representative computed tomography images illustrated the changes in tumor lesions before and after TKI therapy in patients who responded to treatment (Fig. 1b). In the lung (left image) and liver (right image), the outlined tumor boundaries demonstrated a clear reduction in tumor size post-treatment, as indicated by the arrows. Notably, in the right liver lesions, a significant decrease in the viable solid portion was observed after treatment.

### Identification of DEPs based on response to TKI therapy

**Fig. 2:**
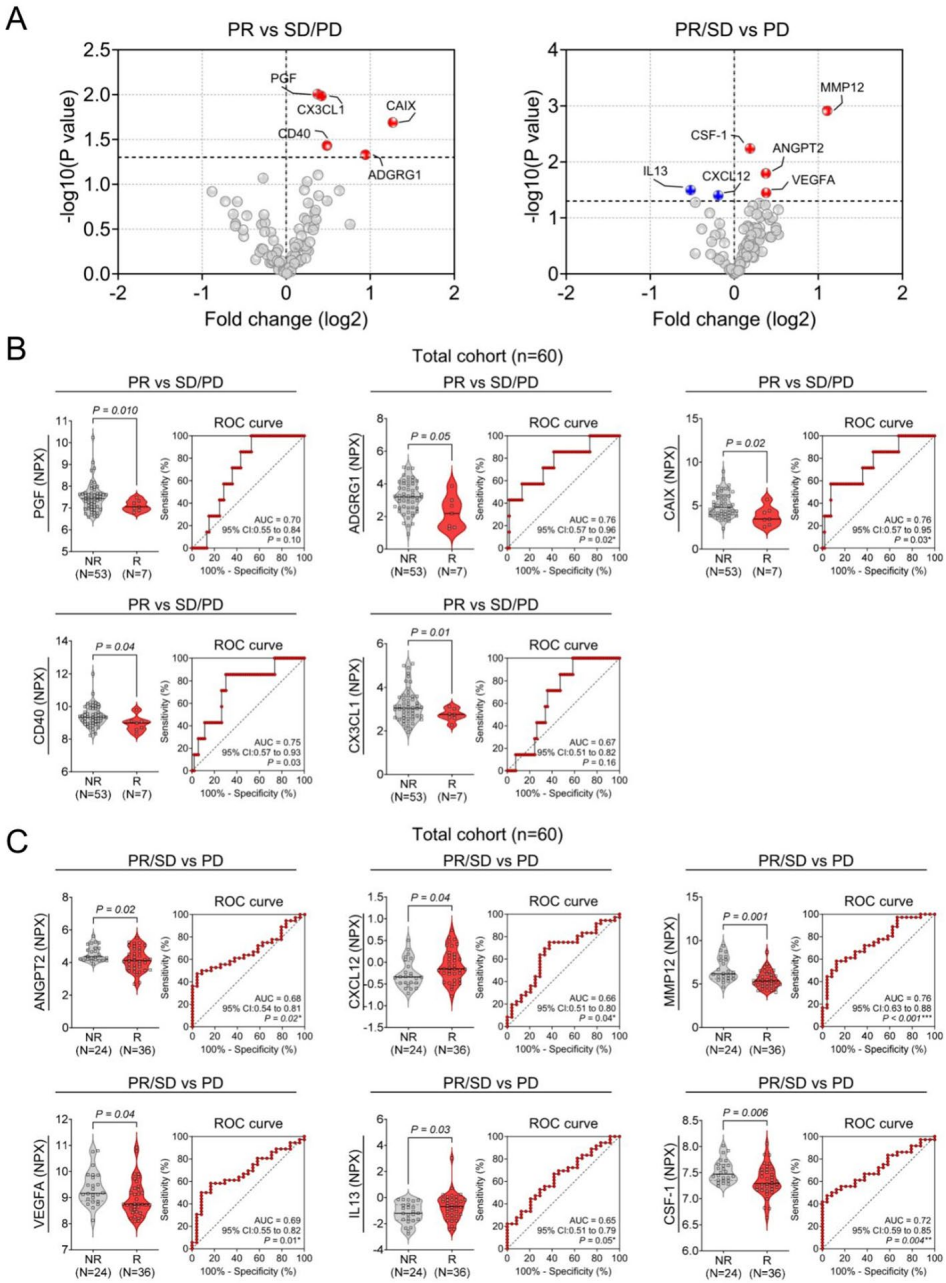
Analysis of differentially expressed proteins (DEPs) and their predictive power in TKI-treated patients with HCC. **a** Volcano plots displaying DEPs between the partial responder (PR) and stable/progressive disease (SD/PD) groups (left) and between the PR/SD and PD groups (right). Proteins with significant fold changes and *P*-values are highlighted in red, including key markers, such as PGF, CX3CL1, CAIX, and MMP-12. **b** Violin plots and ROC curves showing the predictive performance of selected proteins (PGF, ADGRG1, CAIX, CD40, and CX3CL1) in distinguishing PR from SD/PD. The *P*-values indicate the significance of the differences between groups, and ROC curves assess the sensitivity and specificity of each marker. **c** Violin plots and ROC curves showing the predictive capacity of proteins (ANGPT2, CXCL12, MMP-12, VEGFA, IL-13, and CSF-1) to distinguish PR/SD from PD. The area under the curve (AUC) values and confidence intervals are provided for each ROC curve. Statistical significance is denoted as *P < 0.05, **P < 0.01, and ***P < 0.001. HCC, hepatocellular carcinoma; PGF, placental growth factors; CX3CL1, C-X3-C motif chemokine ligand 1; CAIX, carbonic anhydrase IX; MMP-12, matrix metalloproteinase 12; ROC, receiver operating characteristic; ADGRG1, adhesion G protein-coupled receptor G1; CD40, cluster of differentiation 40; ANGPT2, angiopoietin 2; CXCL12, C-X-C motif chemokine ligand 12; VEGFA, vascular endothelial growth factor A; IL-13, interleukin 13; CSF-1, colony-stimulating factor 1; NR, non-responder; R, responder

Figure 2a displays a volcano plot illustrating the DEPs according to treatment response. The proteins highlighted in red and blue indicate statistically significant differential expression. In the left plot of Fig. 2a, five protein–placental growth factors (PGFs), adhesion G protein-coupled receptor G1 (ADGRG1), carbonic anhydrase IX (CAIX), cluster of differentiation 40 (CD40), and C-X3-C motif chemokine ligand 1 (CX3CL1), were identified as significantly upregulated proteins in the SD and PD groups compared with those in the PR group. Figure 2b compares the expression of these five DEPs between the PR and SD/PD groups. Among them, ADGRG1 (AUROC = 0.76, 95% CI: 0.57–0.96, *P* = 0.02) and CAIX (AUROC = 0.76, 95% CI: 0.57–0.95, *P* = 0.03) demonstrated the highest AUROC value. The right plot in Fig. 2a shows a volcano plot illustrating the DEPs between the PR/SD and PD groups. The expressions of angiopoietin 2 (ANGPT2), matrix metalloproteinase 12 (MMP-12), vascular endothelial growth factor A (VEGFA), and colony-stimulating factor 1 (CSF-1) were significantly upregulated, whereas those of IL-13 and C-X-C motif chemokine ligand 12 (CXCL12) were downregulated in the PD group (Fig. 2c). In the ROC analysis, MMP-12 exhibited the highest AUROC value of 0.76 (95% CI: 0.6–0.88, *P* < 0.001, Fig. 2c) among the six DEPs.

**Fig. 3:**
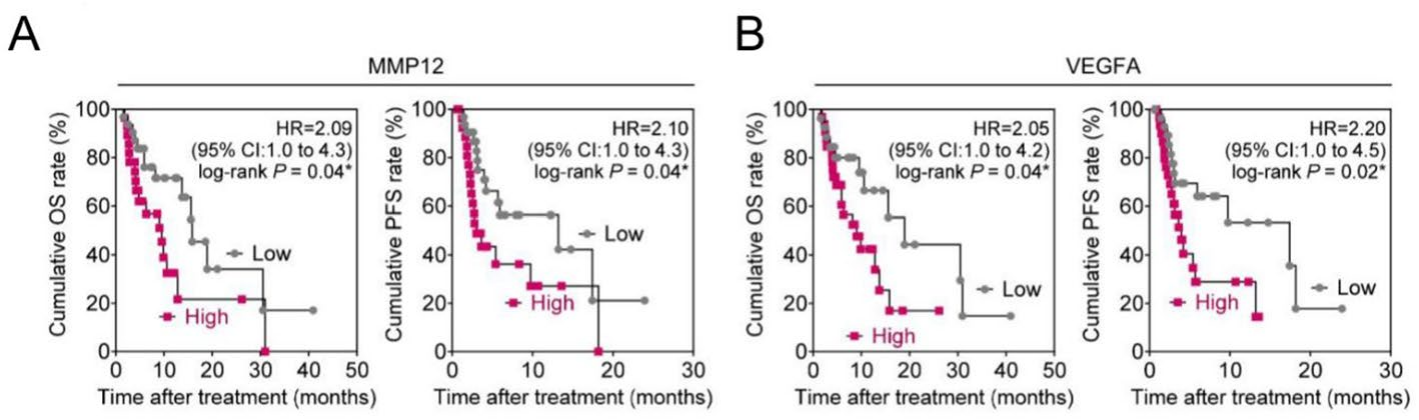
Kaplan–Meier survival curves for MMP-12 and VEGFA expression levels in TKI-treated patients with HCC. **a** Kaplan–Meier survival curve comparing high and low expression of MMP-12 stratified by the median expression level. **b** Kaplan–Meier survival curve comparing high and low expression of VEGFA stratified by the median expression level. Statistical significance was assessed using the log-rank test (*P < 0.05, **P < 0.01, ***P < 0.001). HCC, hepatocellular carcinoma; MMP-12, matrix metalloproteinase 12; VEGFA, vascular endothelial growth factor A

Kaplan–Meier survival analysis was performed for the 11 plasma proteins identified in the DEP analysis (Fig. 3). Patients with higher plasma MMP-12 levels had significantly poorer PFS (hazard ratio, HR = 2.10, 95% CI: 1.0–4.3, *P* = 0.04) and OS (HR = 2.09, 95% CI: 1.0–4.3, *P* = 0.04; Fig. 3a). Patients with elevated plasma VEGFA levels also had significantly shorter PFS (HR = 2.20, 95% CI: 1.0–4.3, *P* = 0.02) and OS (HR = 2.05, 95% CI: 1.0–4.2, *P* = 0.04) than those with lower VEGFA levels (Fig. 3b). In contrast, the remaining nine proteins showed no significant association with PFS or OS. Data for these proteins with non-significant log-rank *P* values are shown in Fig. S1.

### Analysis of the targeted plasma proteome based on the 12-month PFS criterion

**Fig. 4:**
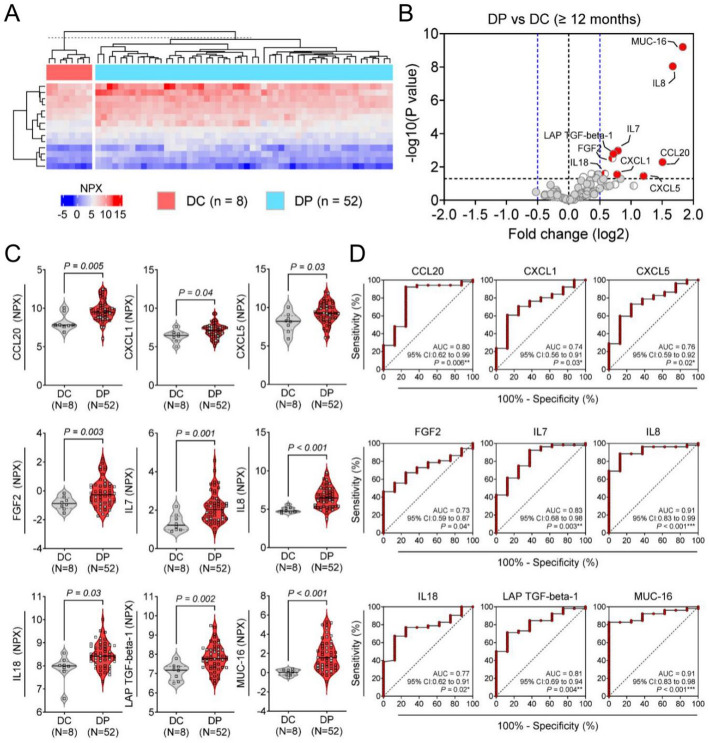
Comparative analysis of selected protein markers between the disease control (DC) and disease progression (DP) groups in TKI-treated HCC patients (PFS ≥ 12 months for DC and PFS < 12 months for DP). **a** Heatmap showing the expression levels of the nine selected proteins between the DC (PFS ≥ 12 months, *n* = 8) and DP (PFS < 12 months, *n* = 52) groups. Higher protein expression is indicated in red, and lower expression in blue. The heatmap reveals distinct clusters based on protein expression (NPX values) between the two groups. **b** Volcano plot showing the log_2_ fold changes and -log_10_ *P*-values of the nine selected proteins between DC and DP groups. Statistically significant proteins are marked in red, indicating their relevance in differentiating between two patient groups. **c** Violin plots comparing the expression levels of the nine selected proteins between DC and DP groups. *P* values indicate the statistical significance of the differences in expression between the two groups. **d** ROC curves displaying the predictive power of the nine selected proteins in distinguishing between DC and DP groups. Higher AUC values indicate better predictive performance for the specific protein markers. Statistical significance is denoted as *P < 0.05, **P < 0.01, and ***P < 0.001. HCC, hepatocellular carcinoma; PFS, progression-free survival; NPX, normalized protein expression; ROC, receiver operating characteristic; AUC, area under the curve; CCL20, C-C motif chemokine ligand 20; CXCL1, C-X-C motif chemokine ligand; CXCL5, C-X-C motif chemokine ligand 5; FGF2, fibroblast growth factor 2; IL-7, interleukin 7; IL-8, interleukin 8; IL-18, interleukin 18; LAP TGF-beta-1, latency-associated peptide transforming growth factor beta 1; MUC-16, mucin 16

Additional analyses were conducted to identify plasma protein biomarkers predictive of disease control with TKI therapy (PFS ≥ 12 months). Based on progression-free survival, patients were categorized into disease control (DC; PFS ≥ 12 months) and disease progression (DP) groups. Patients with PFS ≥ 12 months were analyzed separately to enrich for biomarkers associated with durable response to TKI treatment rather than general tumor prognosis. Among the 60 patients, 8 who achieved a PFS of ≥ 12 months were grouped into the DC group, whereas the remaining 52 were classified as DP. Figure 4a presents a heatmap showing DEPs between the two groups. Hierarchical clustering in the heat map distinctly separated the DC and DP based on NPX levels, highlighting the distinct protein expression patterns between the groups. Figure 4b displays a volcano plot highlighting the differences in pre-treatment plasma protein expression between the two groups. A total of nine proteins, namely C-C motif chemokine ligand 20 (CCL20), C-X-C motif chemokine ligand (CXCL1), C-X-C motif chemokine ligand 5 (CXCL5), fibroblast growth factor 2, IL-7, IL-8, IL-18, latency-associated peptide-transforming growth factor beta 1 (LAP TGF-beta-1), and mucin 16 (MUC-16), were significantly upregulated in the DP group (Fig. 4c). Among these, IL-8 and MUC-16 demonstrated notably high diagnostic performance in distinguishing the DC group, with AUROC values of 0.91 (95% CI: 0.83–0.99, *P* < 0.001 and 95% CI: 0.83–0.98, *P* < 0.001; Fig. 4d).

**Fig. 5:**
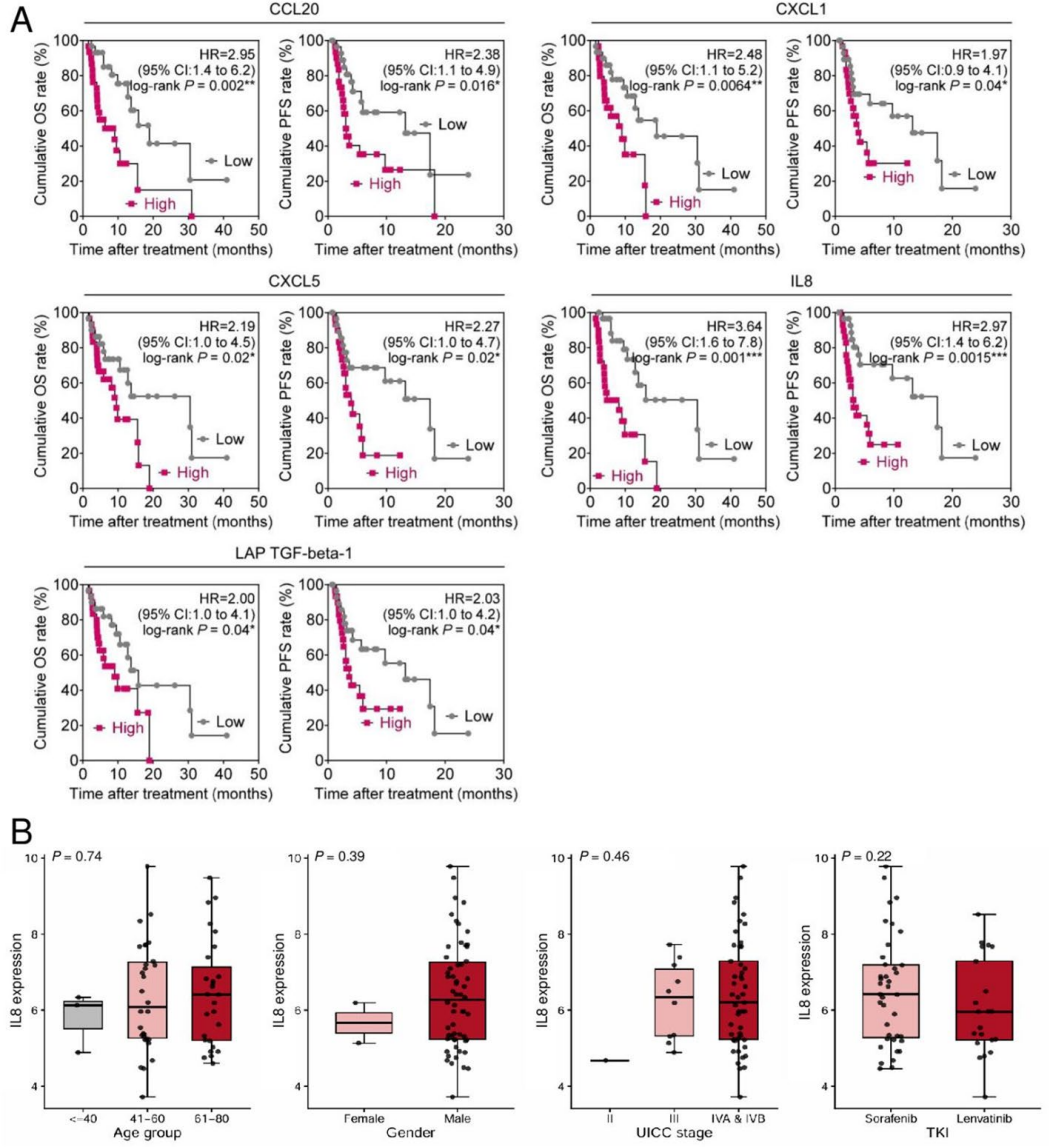
Kaplan–Meier survival curves for significant prognostic markers in TKI-treated patients with HCC. **a** Kaplan–Meier survival curves showing the overall survival of patients stratified by high (pink squares) and low (gray circles) expression levels of key prognostic markers. Each panel represents a different marker, with expression levels divided by the median values. Patients with high expression levels were compared with those with low expression levels, and survival outcomes were compared. The statistical significance of differences in survival between the two groups was assessed using the log-rank test. **b** Stratified analysis of baseline plasma IL-8 levels (NPX) across age, sex, UICC stage, and TKI treatment (lenvatinib vs. sorafenib), showing no significant differences among clinical subgroups (one-way ANOVA or unpaired *t*-test, *P < 0.05, **P < 0.01, ***P < 0.001). HCC, hepatocellular carcinoma; CCL20, C-C motif chemokine ligand 20; CXCL1, C-X-C motif chemokine ligand; CXCL5, C-X-C motif chemokine ligand 5; IL-8, interleukin 8; LAP TGF-beta-1, latency-associated peptide transforming growth factor beta 1; HR, hazard ratio; NPX, normalized protein expression

Survival analysis was performed for nine proteins that showed significant differences between the DC and DP groups (Fig. 5a, Fig. S2). Of these, five proteins (CCL20, CXCL1, CXCL5, IL-8, and LAP TGF-beta-1) were significantly associated with poorer OS and PFS in patients with high expression levels. Among them, IL-8 exhibited the highest HR and lowest *P*-value in OS (HR = 3.64, 95% CI: 1.6–7.8, *P* = 0.001) and PFS (HR = 2.97, 95% CI: 1.4–6.2, *P* = 0.0015), highlighting its potential as a key prognostic biomarker. Plasma IL-8 levels were not significantly different according to age, sex, UICC stage, or TKI treatment (sorafenib vs. lenvatinib), indicating that baseline IL-8 expression is not confounded by major clinical characteristics (Fig. 5b). To explore potential synergistic effects among differentially expressed proteins, additional correlation and multi-marker analyses were performed. Plasma IL-8 levels showed significant positive correlations with MMP12 and VEGFA (Fig. S3a). Receiver operating characteristic analyses demonstrated that combined biomarker models incorporating IL-8 with MMP12 and/or VEGFA achieved high discriminative performance, with AUROC values comparable to or slightly higher than that of IL-8 alone (Fig. S3b). These findings suggest that IL-8 may play a pivotal role in disease progression and may serve as a critical target for therapeutic interventions aimed at improving the clinical outcomes of patients with HCC. To further validate the clinical significance of IL-8 and its role in TME, we conducted additional analyses using publicly available datasets.

### Validation of elevated CXCL8 (IL-8) expression in TKI resistance using public transcriptomic data

**Fig. 6:**
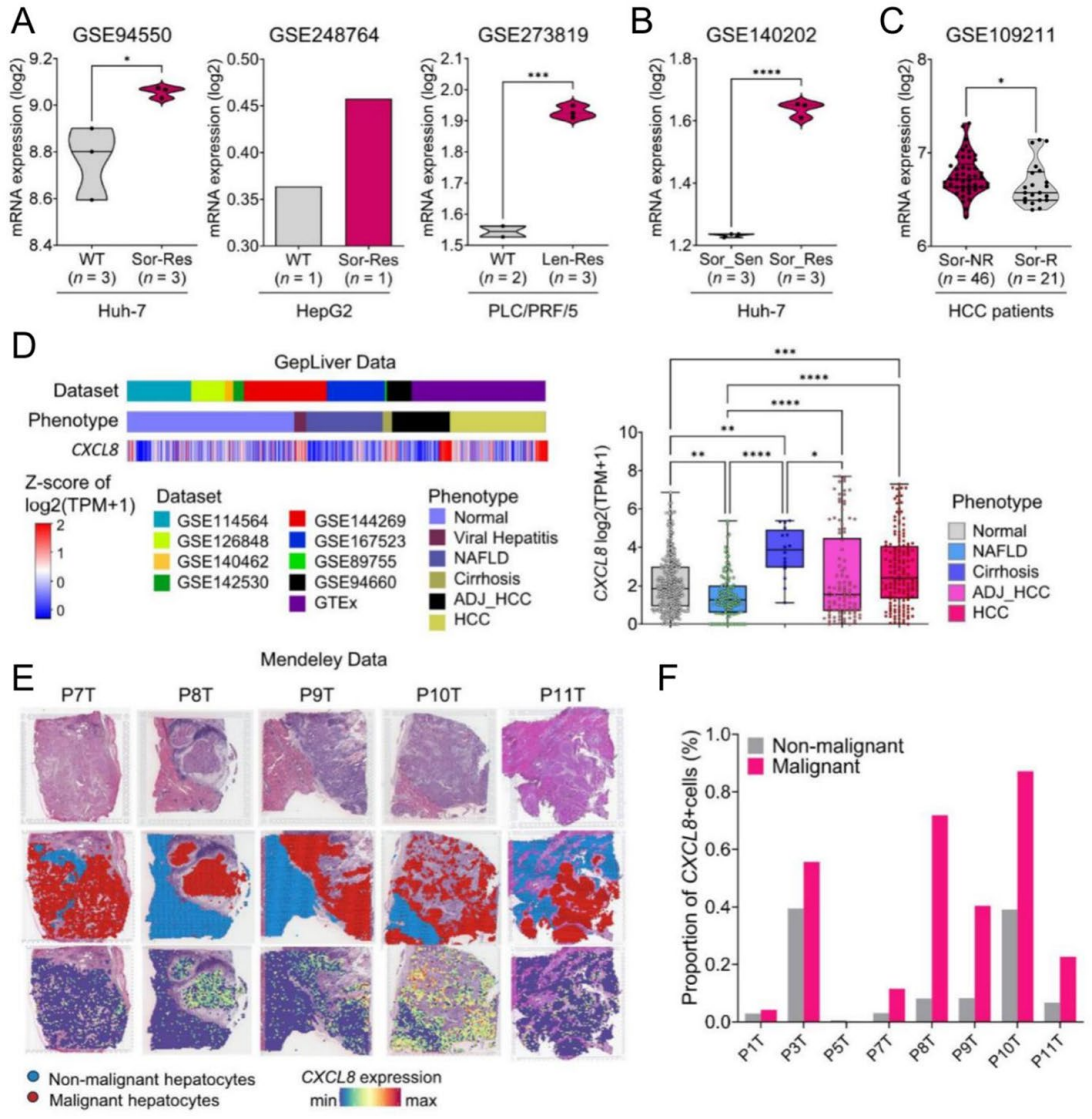
*CXCL8* expression is associated with TKI resistance, HCC progression, and its spatial distribution in malignant and non-malignant hepatocytes. **a** CXCL8 mRNA expression in TKI-treated HCC cell lines (Huh-7, HepG2, and PLC/PRF/5) comparing sorafenib-resistant (Sor-Res) or lenvatinib-resistant (Len-Res) cells with wild-type (WT) cells. **b** CXCL8 mRNA expression in sorafenib-sensitive (Sor-Sen) and Sor-Res Huh-7 cells from the GSE140202 dataset. **c** CXCL8 mRNA expression in the sorafenib non-responder (Sor-NR) and responder (Sor-R) groups from the GSE109211 dataset. **d** CXCL8 mRNA expression across different liver conditions (normal liver, non-alcoholic fatty liver disease, cirrhosis, and HCC) analyzed using integrated GepLiver datasets. **e** Spatial visualization of *CXCL8* expression in malignant and non-malignant hepatocytes from patients with HCC (Mendeley Data) with intensity represented by a color gradient. **f** Proportion of *CXCL8 +* cells in malignant and non-malignant hepatocytes across HCC patient samples. Statistical significance is indicated as *P < 0.05, **P < 0.01, ***P < 0.001, and ****P < 0.0001. HCC, hepatocellular carcinoma; TKI, tyrosine kinase inhibitor; CXCL8 (IL-8), C-X-C motif chemokine ligand 8 (interleukin 8); NAFLD, non-alcoholic fatty liver disease; ADJ_HCC, adjacent HCC; GepLiver, gene expression profile of liver; TPM, transcripts per million

We first examined *CXCL8* (the gene encoding IL-8) expression in response to TKI treatment using public omics datasets. At the cell-line level, we analyzed *CXCL8* expression in relation to TKI resistance. Transcriptomic data from wild-type (WT) and TKI-resistant (Res) HCC cell lines, including Huh7, HepG2, and PLC/PRF/5, were obtained from datasets GSE94550, GSE248764, and GSE273819, respectively (Fig. 6a). Across all three cell lines, a significant increase in *CXCL8* expression was observed in TKI-resistant cells compared with that in WT cells. Additionally, in another cell line dataset, GSE140202, sorafenib-resistant cell lines exhibited markedly higher *CXCL8* expression levels than sorafenib-sensitive cell lines (Fig. 6b). Next, we analyzed *CXCL8* expression in pre-treated HCC tumor tissues from an HCC cohort using the transcriptomic dataset GSE109211. The analysis revealed that *CXCL8* expression was significantly higher in the tumor tissues of sorafenib non-responders than in responders (Fig. 6c). These findings collectively demonstrate that elevated *CXCL8* expression is associated with TKI resistance in both HCC cell lines and the HCC cohort, corroborating the patterns observed in the pre-treatment plasma IL-8 results in our study.

### Exploration of *CXCL8* expression in liver disease and malignant hepatocytes

To further explore the significance of *CXCL8* expression in liver disease and HCC, we analyzed the GepLiver database (DB) and Mendeley DB. These analyses were performed to compare *CXCL8* expression across different liver conditions and to examine its spatial and cellular distribution within HCC tissues, providing insights into its potential role in disease progression and the TME. First, we analyzed *CXCL8* expression under various liver conditions using the GepLiver DB. The analysis revealed significantly higher *CXCL8* expression in HCC tissues than in non-tumor tissues, including normal liver, steatotic liver, and peri-tumoral non-cancerous tissues (Fig. 6d). To further validate these findings, a spatial transcriptomic analysis was performed to examine the distribution of *CXCL8* expression in HCC tissues. Figure 6e shows hematoxylin and eosin-stained sections along with spatial maps of the malignant and non-malignant regions. *CXCL8* expression was predominantly localized in malignant hepatocytes, with higher expression levels concentrated in tumor-dense areas, as visualized using heat maps. Quantification of *CXCL8*-positive cells confirmed this trend, with a significantly higher proportion of *CXCL8*-positive cells observed in the malignant regions than in the non-malignant regions across all analyzed samples (Fig. 6f). Notably, certain samples, such as P8T and P9T, demonstrated more than a four-fold increase in *CXCL8*-positive cell populations within malignant versus non-malignant regions. These results suggest that *CXCL8* expression is elevated in HCC and preferentially localized to malignant regions, highlighting its potential role in tumor progression and its relevance as a biomarker and therapeutic target in HCC.

### Single-cell analysis reveals *CXCL8* expression patterns and its role in the tumor microenvironment of HCC

**Fig. 7:**
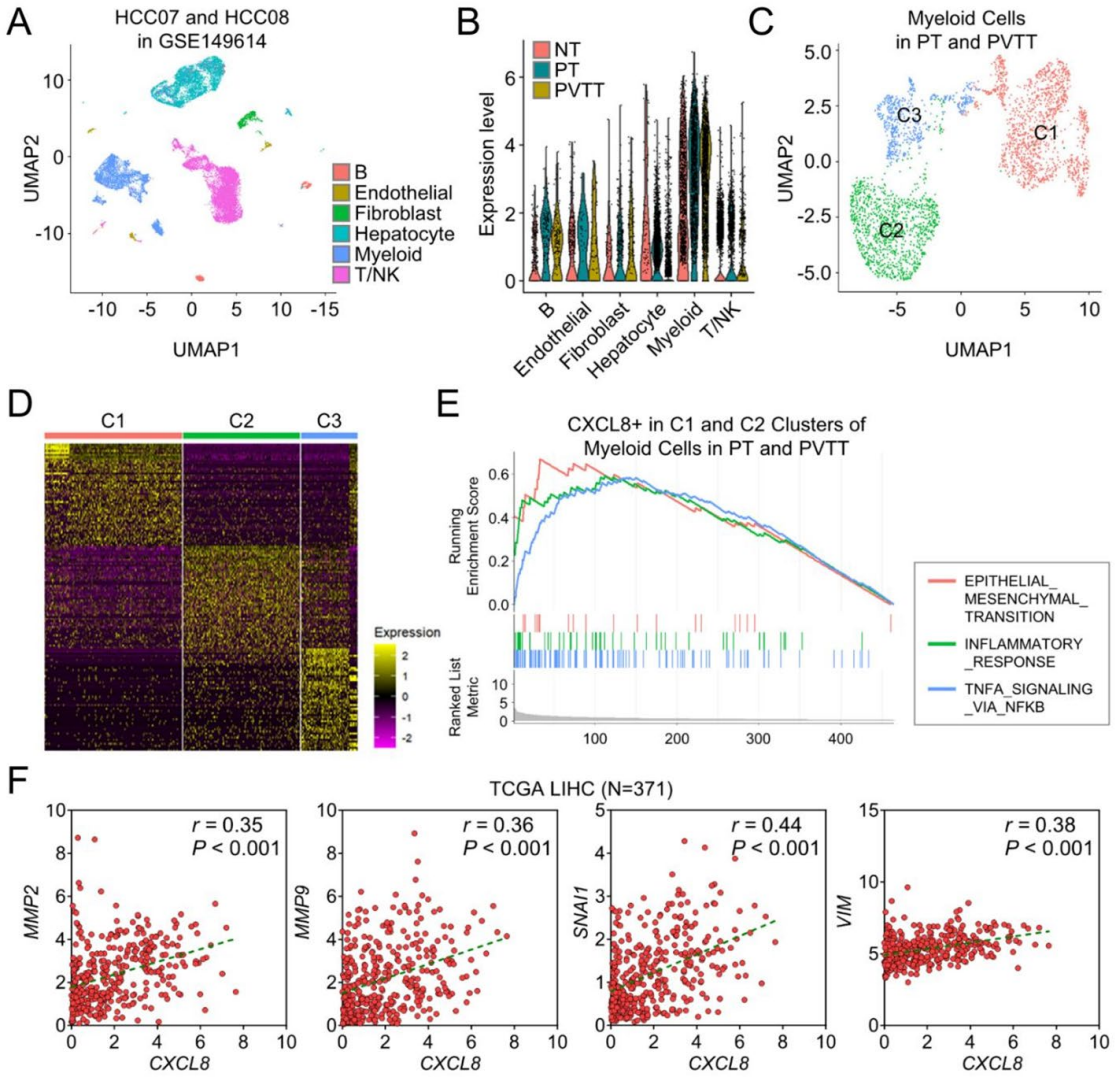
scRNA-seq analysis of *CXCL8* expression in NT, PT, and PVTT samples from the GSE149614 dataset. **a** UMAP plot showing the distribution of all cell types in HCC07 and HCC08 samples from the GSE149614 dataset, with cell types visualized as distinct clusters. **b** Violin plot comparing *CXCL8* expression levels in normal tissue (NT), primary tumor (PT), and portal vein tumor thrombus (PVTT). **c** UMAP plot of myeloid cells in PT and PVTT, classified into three clusters (C1, C2, and C3). **d** Heatmap showing gene expression patterns of *CXCL8* and related genes across C1, C2, and C3 clusters. **e** ssGSEA analysis of *CXCL8*-positive myeloid cells in C1 and C2 clusters, highlighting enrichment of EMT, inflammatory response, and TNF-α signaling via NF-κB pathways. **f** Scatter plots using TCGA data (*N* = 371) showing correlations (*r* > 0.3) between *CXCL8* expression and EMT-related genes. Statistical significance was set at *P* < 0.001. UMAP, uniform manifold approximation and projection; HCC, hepatocellular carcinoma; ssGSEA, single-sample gene set enrichment analysis; TNF-α, tumor necrosis factor-alpha; NF-κB, nuclear factor kappa-light-chain-enhancer of activated B cells

To further elucidate the cellular origins and functional implications of *CXCL8* expression in HCC, we analyzed single-cell RNA sequencing data from the GSE149614 dataset, which includes HCC and surrounding non-tumor tissues, enabling the identification of specific cell types that contribute to *CXCL8* expression. Figure 7a shows the UMAP visualization of cell clusters identified from HCC and adjacent non-tumor tissues, including hepatocytes, myeloid cells, endothelial cells, fibroblasts, B cells, and T/NK cells. Myeloid cells demonstrated the highest *CXCL8* expression levels, as highlighted in the violin plot (Fig. 7b). To further refine the cellular origin of CXCL8, we analyzed an independent publicly available GepLiver dataset with detailed cell-type annotations. As shown in Supplementary Fig. S4a–c, CXCL8 expression was enriched in tumor-associated myeloid compartments. Sub-clustering of myeloid populations revealed predominant CXCL8 expression in neutrophil-dominant subsets and selected monocyte/macrophage clusters (Fig. S4d–f), further clarifying the myeloid subtype–specific distribution of CXCL8 in HCC. To investigate the functional heterogeneity within myeloid cells, a subset of myeloid cells from PTs and PVTT was recruited. This analysis revealed three distinct clusters: C1, C2, and C3 (Fig. 7c). Differential gene expression analysis between these clusters identified *CXCL8* as a marker that was highly enriched in the C1 and C2 clusters (Fig. 7d). GSEA of *CXCL8* + myeloid cells in clusters C1 and C2 revealed significant enrichment in pathways associated with EMT, inflammatory responses, and TNF-α signaling via NF-κB, suggesting a role of *CXCL8* in promoting tumor progression and immune modulation (Fig. 7e). Finally, we validated these findings using TCGA_LIHC data. *CXCL8* expression was positively correlated with genes associated with EMT (e.g., *SNAI1*, *VIM*) and matrix remodeling (e.g., *MMP-2*, *MMP-9*) (Fig. 7f). These results further support the association between *CXCL8* expression and the pathways driving tumor invasiveness and immune evasion in HCC.

## Discussion

In this study, we performed a targeted proteomic analysis using a 96-protein immuno-oncology panel to identify plasma biomarkers predictive of therapeutic responses to TKI therapy in patients with advanced HCC. Through the analysis of pre-treatment blood samples, we identified multiple differentially expressed plasma proteins associated with treatment response and clinical outcomes. Among these, IL-8 stood out as a key biomarker, demonstrating significant predictive power for distinguishing disease control from disease progression and showing strong correlations with shorter PFS and OS. These findings were further supported by analyses of public transcriptomic datasets, which consistently showed higher *CXCL8* expression in TKI-resistant HCC cell lines and tumor tissues from TKI-treated HCC cohorts. Additionally, *CXCL8* expression was markedly elevated in HCC tissues compared with that in other liver conditions, as revealed by GepLiver and spatial transcriptomic analyses. Single-cell RNA sequencing data provided deeper insights, showing that *CXCL8* was predominantly expressed in myeloid cells within the TME, particularly in clusters enriched for pathways associated with EMT, inflammatory responses, and TNF-α signaling. Collectively, these results highlight the significant potential of IL-8 as a robust prognostic biomarker and promising therapeutic target in advanced HCC.

The identification of predictive biomarkers for TKI therapy in HCC remains a challenge, hindering the improvement of therapeutic outcomes. Owing to the challenges in obtaining tumor tissues, the exploration of blood biomarkers has attracted attention as a noninvasive alternative for predicting TKI resistance in HCC. Plasma biomarkers reflect systemic changes and TME dynamics, offering practical advantages. Circulating VEGF, ANGPT2, and IL-6 levels have been associated with poor survival and resistance in patients with TKI-treated HCC [30–32]. Additionally, plasma secreted phosphoprotein 1 and risk models incorporating hepatocyte growth factor and fibroblast growth factor have shown promise in predicting treatment responses and survival [33, 34]. Although several previously reported circulating markers, such as IL-6 and VEGF, were included in the analysis, IL-8 demonstrated the strongest predictive performance among the analyzed proteins. IL-8 showed the highest discriminatory power for treatment response and the most consistent associations with progression-free and overall survival. While IL-6 and VEGF are largely linked to systemic inflammation and angiogenesis, their predictive value for TKI-specific response appears to be context-dependent. In contrast, IL-8 is closely associated with myeloid-driven immunosuppression, EMT, and resistance-related signaling pathways, which may explain its higher predictive specificity in our cohort.

In this study, IL-8 emerged as a pivotal plasma biomarker with significant predictive power for distinguishing disease control from disease progression in patients with advanced HCC undergoing TKI therapy. As a key inflammatory cytokine, IL-8 is known to modulate inflammatory responses [35]. Previous studies have demonstrated that IL-8 is positively correlated with the expression of drug resistance genes in human hepatoma cells and HCC tissues, which is closely associated with reduced responses to anticancer therapy [36, 37]. Beyond its role in treatment resistance, IL-8 expression has been reported to increase with disease progression across chronic liver diseases, including cirrhosis and hepatocellular carcinoma, compared with normal liver tissue [38]. Consistent with these observations, tissue-level analyses using the GepLiver database revealed that IL-8 expression was significantly higher in HCC tumor tissues than in normal liver, steatotic liver, or adjacent non-tumorous tissues. At the treatment-response level, public transcriptomic datasets have consistently demonstrated higher *CXCL8* expression in TKI-resistant HCC cell lines and tumor tissues from TKI-treated HCC cohorts. Spatial and single-cell transcriptomic analyses further revealed that *CXCL8* was predominantly expressed in myeloid cells within the TME, particularly in clusters linked to EMT, inflammatory responses, and TNF-α signaling. Previous studies have highlighted the interplay of TME cells, including MDSCs, in TKI resistance and the critical role of IL-8 in the TME [39–41]. Beyond IL-8–specific mechanisms, inflammatory cytokines have been shown to exert complex and context-dependent effects on hepatic tumor microenvironment remodeling and immune regulation in HCC [42]. Importantly, a preclinical study showed that targeting tumor-infiltrating Ly6G+ myeloid cells significantly enhances the efficacy of sorafenib in a mouse model of HCC [43]. IL-8 has been implicated in the promotion of angiogenesis, immune suppression, and tumor invasiveness, correlating with poor responses to certain therapies, such as sorafenib [40]. The signaling pathways recruit immunosuppressive cells, such as MDSCs, and activate NF-κB and STAT3, thereby driving tumor progression [40, 44–46]. Collectively, these findings indicate that IL-8 could be an informative biomarker for predicting response to TKI treatment and a potential therapeutic target. Although IL-8 may also reflect general tumor aggressiveness and therefore carry prognostic implications in HCC, the primary objective of this study was to identify biomarkers predictive of treatment response rather than markers of baseline disease severity. To disentangle predictive effects from general prognosis, we performed subgroup analyses in patients with prolonged progression-free survival (PFS ≥ 12 months), thereby reducing the influence of rapidly progressive disease and enriching for biomarkers associated with sustained therapeutic benefit. Importantly, discrepancies in predictive markers between whole-cohort analyses and the prolonged-PFS subgroup likely arise from differences in clinical context, as whole-cohort analyses may capture early progression driven by intrinsic tumor aggressiveness. Furthermore, given that the median PFS of first-line TKIs in advanced HCC is substantially shorter—approximately 3–4 months for sorafenib and 7 months for lenvatinib—a threshold of ≥ 12 months was selected to define a clinically meaningful subset characterized by durable disease control rather than transient stabilization.

This study has some limitations that warrant consideration. First, this study was conducted at a single center in an HBV-endemic region and involved a relatively small sample size, which may limit the generalizability of the findings to HCC populations with different etiological backgrounds or geographic regions. Second, the findings were not validated in a large prospective cohort, which restricts the generalizability of the results. Future studies with diverse and larger patient populations are required to confirm the predictive value of IL-8 and other biomarkers. Third, although predictive biomarkers may differ between sorafenib and lenvatinib due to their distinct molecular targets and mechanisms of action, we were unable to perform drug-specific subgroup analyses because the sample size within each treatment group (sorafenib, *n* = 39 and lenvatinib, *n* = 21) was insufficient to support statistically robust stratified analyses. Future studies with larger, drug-specific cohorts will be required to determine whether distinct predictive biomarkers exist for individual TKIs. Fourth, while IL-8 has been identified as a potential therapeutic target, this study lacks preclinical experiments showing that targeting MDSC-derived IL-8 can overcome TKI resistance in HCC. Such functional studies are essential for establishing causality and evaluating therapeutic feasibility.

In addition, several practical challenges remain for the clinical translation of IL-8 as a routine biomarker. IL-8 detection currently relies on immunoassay-based platforms, which may be affected by inter-assay variability, limited standardization across laboratories, and differences in sample processing. Furthermore, detection cost, turnaround time, and the lack of harmonized cutoff values may limit the immediate implementation of IL-8 as a routine predictive biomarker in clinical practice. Therefore, large-scale, multi-center validation studies and further clinical exploration of IL-8–targeted strategies in combination with TKI therapy will be essential for successful clinical translation. Additionally, this study focused on plasma biomarkers, which provide a minimally invasive approach but may not fully reflect the complexity of intratumoral heterogeneity or local TME dynamics. The incorporation of tissue-based analyses with plasma biomarkers may enhance our understanding of resistance mechanisms. Moreover, the lack of longitudinal sampling limits insight into the temporal dynamics of IL-8 expression during therapy, which may further refine its utility as a predictive biomarker. Finally, although associations with several pathways, such as EMT and inflammatory responses, were identified, a detailed exploration of downstream IL-8 signaling was beyond the scope of this study. Addressing these limitations in future research is critical for translating these findings into clinical practice.

Nevertheless, this study represents a comprehensive effort to identify plasma biomarkers that are predictive of TKI response in advanced HCC. By leveraging a 96-protein immuno-oncology panel, we employed a high-throughput approach to uncover biomarkers, such as IL-8, which demonstrated robust predictive power for distinguishing disease control from disease progression and significant associations with PFS and OS. The integration of multiple datasets, including public transcriptomic, spatial transcriptomic, and single-cell RNA sequencing data, reinforces the reliability of our findings and provides mechanistic insights into the role of IL-8 in TKI resistance and tumor progression.

In conclusion, this study highlights the clinical utility of plasma-based biomarker panels as a noninvasive and practical alternative to tissue-based diagnostics. Despite existing limitations, such as the need for validation in larger cohorts and preclinical models, our study highlights the potential of IL-8 as a predictive biomarker and a potential therapeutic target. Future studies focusing on IL-8-targeted interventions could lead to more personalized and effective treatments, ultimately improving outcomes in patients with advanced HCC.

## Supplementary Information

Below is the link to the electronic supplementary material.

Supplementary Material 1.

## Data Availability

Public transcriptomic datasets analyzed in this study are available from the Gene Expression Omnibus (GEO) under accession numbers GSE94550, GSE248764, GSE273819, GSE140202, GSE109211, and GSE149614. The GepLiver database was used for liver expression profiling. Spatial transcriptomic data were obtained from Mendeley Data (repository ID: skrx2fz79n). Additional data supporting the findings of this study are included in the article and its Supplementary Information; further information is available from the corresponding authors upon reasonable request.
