# Supplementary material for: Plasma interleukin-8 as a predictive biomarker for tyrosine kinase inhibitor response in advanced hepatocellular carcinoma
Source: Discov Oncol. 2026 May 11;17:978. doi: 10.1007/s12672-026-05055-4 (PMC13332102; doi:10.1007/s12672-026-05055-4)
Supplement: Supplementary file 1 — Supplementary Material 1. [file 12672_2026_5055_MOESM1_ESM.pdf]

Table S1. Baseline characteristics of 60 patients with advanced hepatocellularcarcinoma (HCC) who received sorafenib or lenvatinib treatment in the AJOU-HCC cohort.

Characteristics	All Patients (n=60)
Age [years, median (range)]	59 (37-80)
Male sex [n (%)]	58 (95.1)
Treatment modality [n (%)]	
Sorafenib	39 (63.9)
Lenvatinib	21 (34.4)
Platelet ($\times10^9$/L, mean ± SD)	185. 3 ± 93.2
Albumin (g/L, mean ± SD)	4.0 ± 0.5
Bilirubin (mg/dL, mean ± SD)	1.1 ± 1.0
Creatinine (mg/dL, mean ± SD)	1.1 ± 1.5
AFP (ng/L, mean ± SD)	6322.7 ± 15569.5
ALT (U/L, mean ± SD)	58.3 ± 74.7
AST (U/L, mean ± SD)	118.1 ± 250.5
INR (mean ± SD)	1.2 ± 0.5
Cirrhosis, n (%)	54 (90.0)
Underlying liver disease [n (%)]	
CHB	41 (68.3)
CHC	4 (6.7)
Alcoholic	10 (16.7)
Unknown	5 (8.3)
Follow-up period [months median (range)]	9 (1–40)
ECOG performance status [n (%)]	
0	20 (37.0)
1	34 (63.0)
Tumor size (cm, mean ± SD)	11.1 ± 4.7
Modified UICC stage [n (%)]	
II	1 (1.7)
III	10 (16.7)
IVa	32 (53.3)

IVb	17 (28.3)
Responder classification [n (%)]	
CR	0 (0.0)
PR	7 (11.7)
SD	29 (48.3)
PD	24 (40.0)

AFP, alpha-fetoprotein; ALT, alanine aminotransferase; AST, aspartate aminotransferase; INR, international normalized ratio; CHB, chronic hepatitis B; CHC, chronic hepatitis C; ECOG, European Cooperative Oncology Group; UICC, Union for International Cancer Control, CR; Complete response, PR; Partial response, SD; Stable disease, PD; Progressive disease

Table S2. List of abbreviations defined for this manuscript

Abbreviation	Full Term
ANGPT2	Angiopoietin 2
AUROC	Area under the ROC curve
CSF-1	Colony-stimulating factor 1
CR	Complete response
CIs	Confidence intervals
CXCL12	C-X-C motif chemokine ligand 12
DB	Database
DC	Disease control
DEPs	Differentially expressed proteins
DP	Disease progression
EMT	Epithelial-mesenchymal transition
GSEA	Gene set enrichment analysis
HR	Hazard ratio
HCC	Hepatocellular carcinoma
ICI	Immune checkpoint inhibitor
IL-13	Interleukin 13
MMP12	Matrix metalloproteinase 12
mUICC	Modified Union for International Cancer Control
NT	Normal tissue
NF	Nuclear factor
OS	Overall survival
PR	Partial response
PVTT	Portal vein tumor thrombus
PT	Primary tumor
PFS	Progression-free survival
PD	Progressive disease
ROC	Receiver operating characteristic
scRNA-seq	Single-cell RNA sequencing
SD	Stable disease
TME	Tumor microenvironment
TNF	Tumor necrosis factor
TKIs	Tyrosine kinase inhibitors
UMAP	Uniform manifold approximation and projection
VEGFA	Vascular endothelial growth factor A

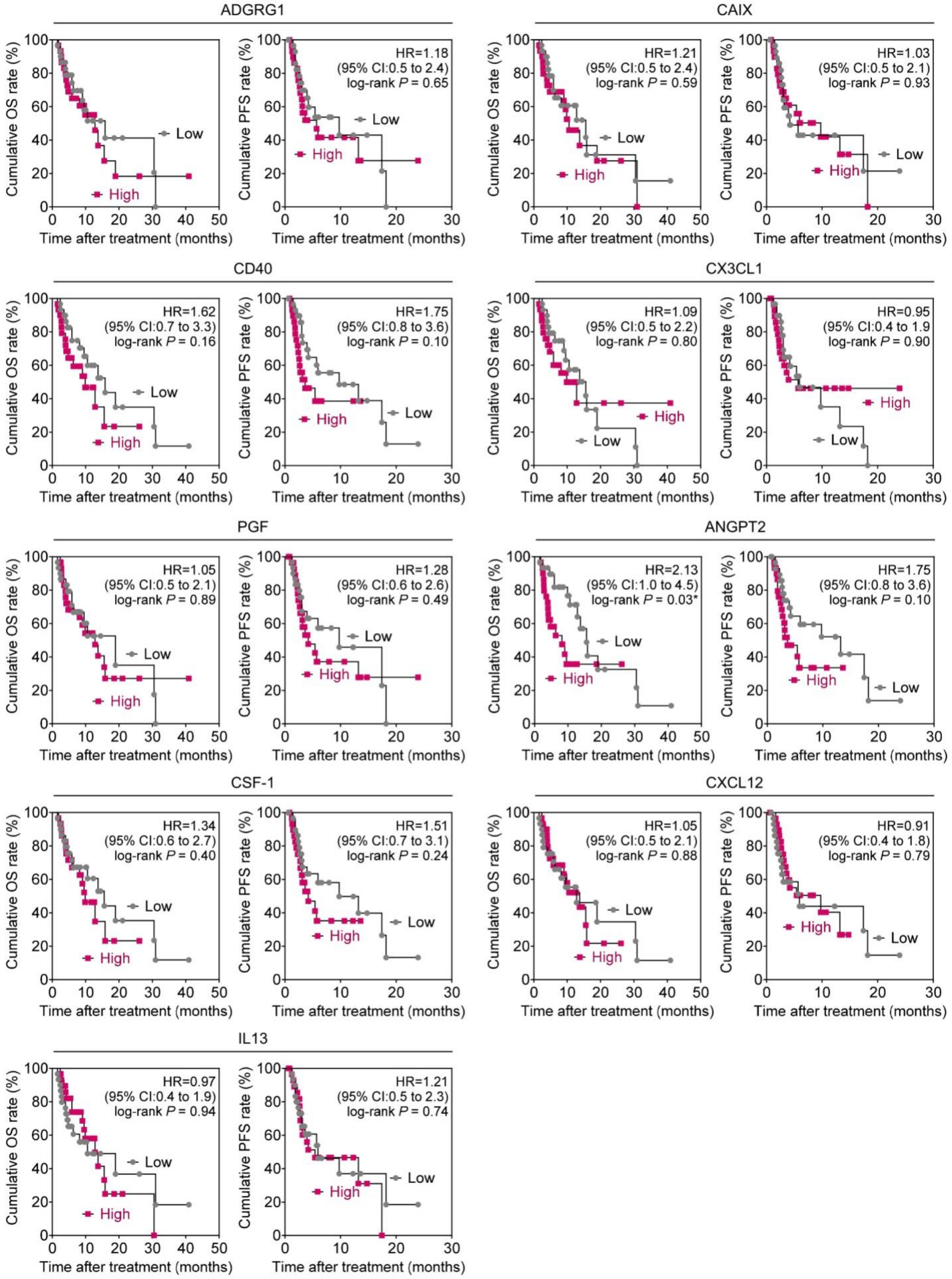

Figure S1. Kaplan-Meier survival analysis of nine protein markers in patients with HCC who were treated with TKIs on response.

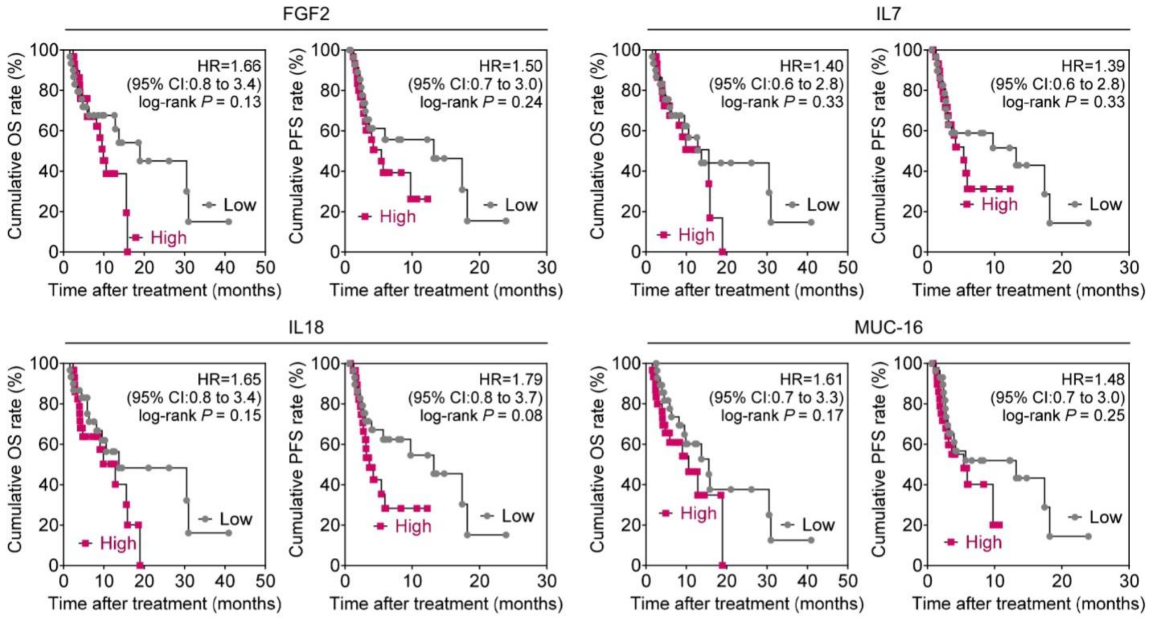

Figure S2. Kaplan-Meier survival analysis for prognostic protein markers in patients with HCC who were treated with TKIs based on the 12-month PFS criterion.

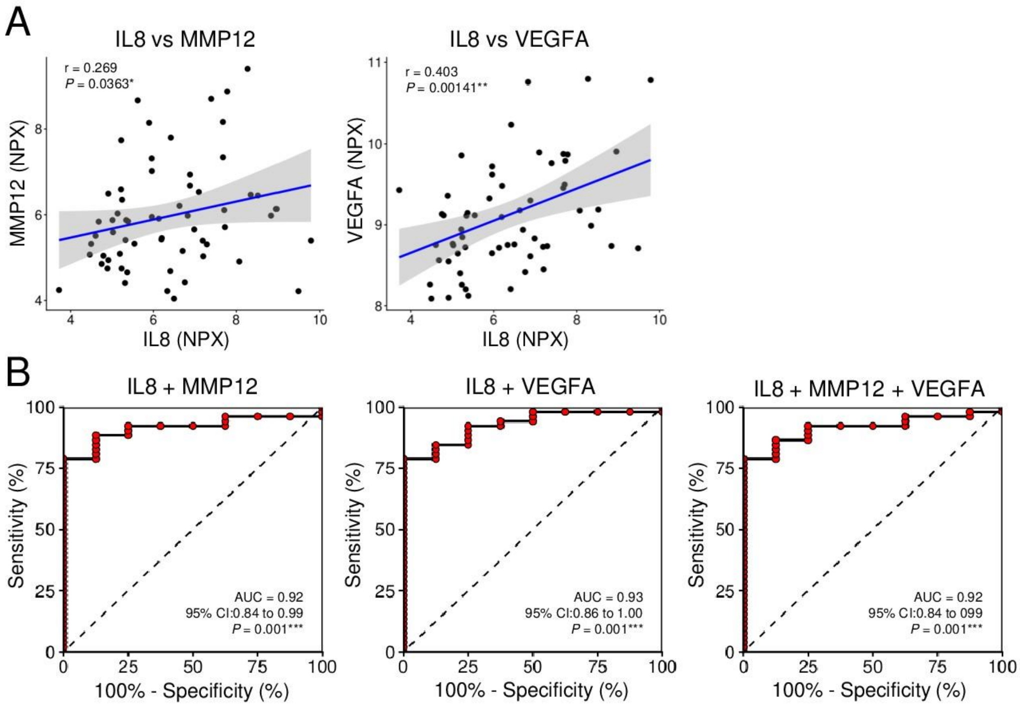

Figure S3. Correlation and multi-marker predictive analyses involving IL-8, MMP12, and VEGFA. (a) Positive correlations between plasma IL-8 levels and MMP12 or VEGFA (NPX values). (b) Receiver operating characteristic (ROC) analyses of combined biomarker models (IL-8 + MMP12, IL-8 + VEGFA, and IL-8 + MMP12 + VEGFA).

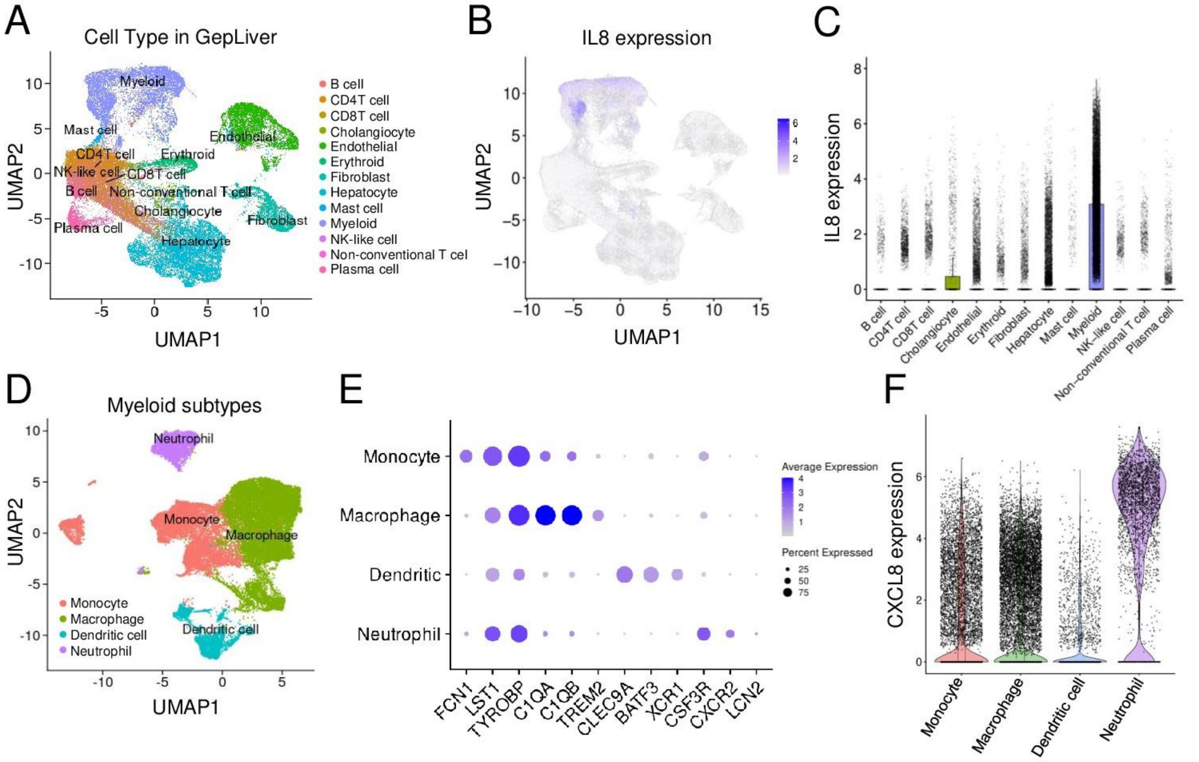

Figure S4. Cell type–specific expression patterns of *CXCL8* in the GepLiver single-cell RNA-seq dataset. (a) UMAP visualization of major cell types in the GepLiver HCC dataset. (b) UMAP feature plot showing CXCL8 (IL-8) expression distribution. (c) CXCL8 expression levels across major cell types. (d) UMAP visualization of myeloid cell subtypes. (e) Dot plot of representative marker genes across myeloid subtypes. (f) CXCL8 expression levels across myeloid subtypes.
